# A Charge-Transfer Salt Based on Ferrocene/Ferrocenium Pairs and Keggin-Type Polyoxometalates

**DOI:** 10.3390/molecules23123150

**Published:** 2018-11-30

**Authors:** Beñat Artetxe, Amaia Iturrospe, Pablo Vitoria, Estibaliz Ruiz-Bilbao, José S. Garitaonandia, Juan M. Gutiérrez-Zorrilla

**Affiliations:** 1 Departamento de Química Inorgánica, Facultad de Ciencia y Tecnología, Universidad del País Vasco UPV/EHU, P.O. Box 644, 48080 Bilbao, Spain; pablo.vitoria@ehu.es (P.V.); estibaliz.ruiz@ehu.eus (E.R.-B.); juanma.zorrilla@ehu.es (J.M.G.-Z.); 2 Centro de Física de Materiales CFM (CSIC–UPV/EHU), Paseo Manuel Lardizabal 5, 20018 Donostia-San Sebastián, Spain; amaia.iturrospe@ehu.eus; 3 Departamento de Física Aplicada II, Facultad de Ciencia y Tecnología, Universidad del País Vasco UPV/EHU, P. O. Box 644, 48080 Bilbao, Spain; js.garitaonandia@ehu.eus; 4 BCMaterials, Basque Center for Materials, Applications and Nanostructures, UPV/EHU Science Park, 48940 Leioa, Spain

**Keywords:** polyoxometalates, ferrocene, single-crystal x-ray diffraction, mössbauer spectroscopy

## Abstract

A new hybrid inorganic-organometallic salt has been obtained from the reaction of the Keggin-type silicotungstate anion with ferrocene in a water/methanol mixture as a result of the partial oxidation of ferrocene molecules to ferrocenium cations. Single-crystal X-ray diffraction analysis reveals the presence of four ferrocenium (Fe^III^) cations and one ferrocene (Fe^II^) molecule per plenary Keggin anion in the crystal structure of [Fe^III^ (Cp)_2_]_4_[SiW_12_O_40_]·[Fe^II^(Cp)_2_]·2CH_3_OH (**1**). Compound **1** thus constitutes the first example in the literature in which ferrocenium and ferrocene species coexist in the structure of a polyoxometalate-based salt. The two crystallographically independent ferrocenium species in the asymmetric unit of **1** exhibit different configurations: One displays an eclipsed conformation with ideal *D_5h_* symmetry, whereas the conformation in the other one is staggered *D_5d_*. The crystal packing of **1** can be best described as an organometallic sub-lattice of ferrocenium and ferrocene species linked by a network of π-π interactions that generates rectangular cavities of about 14 × 10 Å in which strings of Keggin anions and methanol molecules are hosted, further connected to each other via weak O_POM_···C_MeOH_-O_MeOH_···O_POM_ type hydrogen bonds. The charge-transfer nature of the salt has been studied by solid-state diffuse reflectance UV-Vis spectroscopy and the presence of magnetically isolated Fe^III^/Fe^II^ centres has been confirmed by Mössbauer spectroscopy. A topological study carried out on all of the pristine ferrocenyl species deposited in the Cambridge Structural Database (CSD) has allowed two main conclusions to be drawn: (1) these species tend to adopt extreme conformations (either eclipsed or staggered) with less than a 15% of examples showing intermediate states and (2) the oxidation state of the iron centres can be unequivocally assigned on the basis of a close inspection of the Fe···Cp distances, which allows ferrocene neutral molecules and ferrocenium cations to be easily distinguished.

## 1. Introduction

Polyoxometalates (POM) are anionic metal-oxide clusters with rich structural and electronic variety and applications in areas of current interest such as catalysis, nanotechnology, materials science and biomedicine [1]. One of the most relevant properties of POMs is represented by their electron acceptor capability, which can be nicely exemplified by the super-reduced Keggin-type [PMo_12_O_40_]^27−^ cluster reported by Nishimoto et al. for the preparation of molecular cluster batteries and capacitors [2]. Due to their high negative charges and large sizes, POMs can be used as acceptors in the formation of charge-transfer (CT) salts with organic and/or organometallic electron donors. The first example of such compounds was reported in 1988 and included the tetrathiafulvalene (TTF) organic donor in combination with the Keggin-type tungstophosphate anion as acceptor unit [3]. These studies were later extended to Keggin anions with different heteroatoms [4] or transition metal substitutions [5] and to some other POM clusters with different topologies, such as the hexametalate [M_6_O_19_]^2−^ anions. [6] In regard to the organic donor molecules, distinct TTF-like derivatives have also been used in the construction of this type of CT salts, such as bis(ethylenedithio)-tetrathiofulvalene (BEDT-TTF) [7] and tetramethyltetraselenafulvalene (TMTSF) [8]. These studies were mainly focused on the preparation of hybrids with a mixed-valence state on both the organic and inorganic components in order to obtain materials with interesting conducting and magnetic properties [9].

Besides TTF-like groups, organometallic cations, such as ferrocenium [Fe^III^ (C_5_H_5_)_2_]^+^·(Fc^+^) and its derivatives, have also been thoroughly combined with POM clusters to prepare donor-acceptor hybrid materials, because the easily accessible reduction potential of these organometallic species makes them suitable for the formation of radical cations [10]. Ferrocenyl species have attracted great interest in the field of molecular magnetism since the first molecule-based ferromagnet was reported in 1985, namely the [Fe{C_5_(CH_3_)_5_}]·[TCNE] salt based on decamethylferrocenium cations (Fc*^+^) and tetracyanoethylene anions [11,12]. Throughout the last three decades, several ferroceniun-POM salts have been synthesized targeting outer sphere CT molecule-based magnets, in which the interaction between donors and acceptors is based on electrostatic forces and hydrogen bond formation [13]. The structural analysis of such compounds has been revealed as a key factor for the identification of potential interesting properties and therefore, an extensive work has been dedicated to the single-crystal structure resolution of compounds combining Fc^+^ or Fc*^+^ units with POM clusters of very different nature. These include fully-oxidized or partially reduced [H_x_XM_12_O_40_]^n−^ Keggin-type plenary polyoxomolybdates (X = Si, Ge, P, As; H = 0–1) [14,15,16,17] or –tungstates (X = Si, Fe^III^; H = 0–1) [17], first-row transition metal mono-substituted [HPCu(H_2_O)W_11_O_39_]^4−^ Keggin or [Cr(OH)_6_Mo_6_O_18_]^3−^ Anderson–Evans paramagnetic anions [18] and redox-active [HS_2_Mo_18_O_62_]^5−^ Wells–Dawson-type species [19]. Some authors have opted for the organic derivatization of ferrocene units with positively-charged functional groups to lead to ammonium [20] or phosphonium [21] cations that can act as acceptors in the formation of CT salts with Keggin [22] or [M_6_O_19_]^2−^ Lindqvist–type POMs (M = Mo, W) [23]. The covalent linkage of the ferrocene groups to the POM cluster skeleton has also been achieved through the replacement of shell O atoms with ferrocenyl-containing N- or O-donor ligands, as exemplified by some ferrocenylimido derivatives of Lindqvist-type molybdates [24,25] or tris(alkoxo)-capped hexavanadates bearing ferrocene-like substituents [26].

Despite the varied examples of compounds combining ferrocenyl units and POM clusters that can be found in the literature, to date there is no structural evidence of both ferrocenium cations and ferrocene neutral species coexisting in the crystal packing of any POM-based salt. Herein we report the synthesis and structural characterization of compound [Fe(Cp)_2_]_4_[SiW_12_O_40_]·[Fe(Cp)_2_]·2CH_3_OH (**1**) containing ferrocenyl units with both Fe^III^ and Fe^II^ centres as assessed by Mössbauer spectroscopy analyses. The CT nature of the salt has been confirmed by diffuse-reflectance UV-Vis spectroscopy and the topological study carried out using all of the entries deposited in the Cambridge Structural Database (CSD) [27] that contain isolated, pristine ferrocenyl species has allowed to easily discriminate between ferrocenium cations (Fe^III^) and ferrocene molecules (Fe^II^) in the title compound upon close inspection of the Fe-C bond lengths, as well as to establish that this type of organometallic species tend to adopt extreme conformations (either eclipsed or staggered) as observed for **1**.

## 2. Results and Discussion

### 2.1. Synthesis and Infrared Spectroscopy

**Figure 1:**
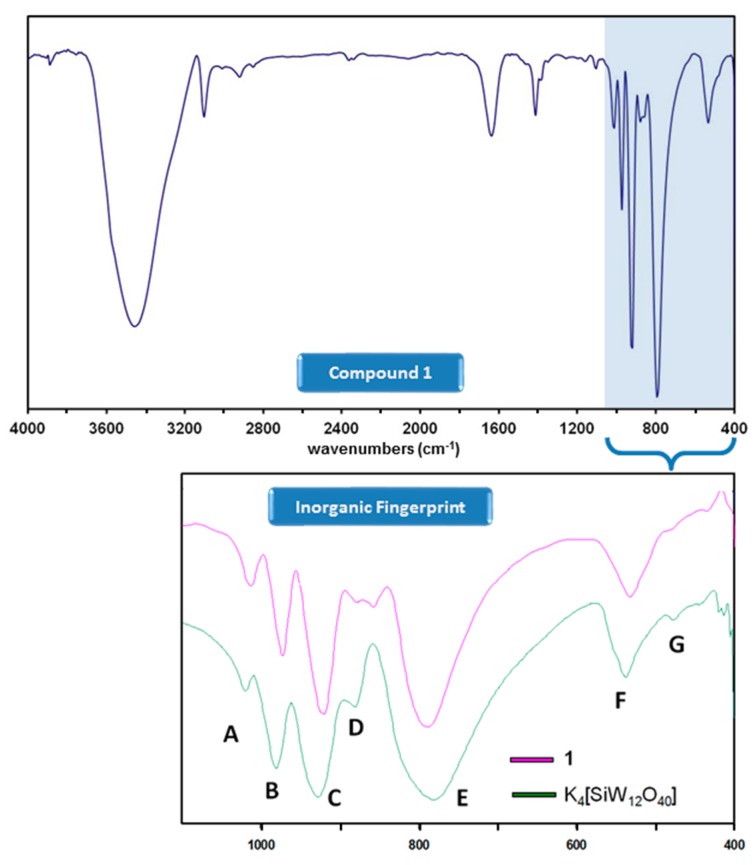
FT-IR spectrum of **1** and comparative detail of the inorganic fingerprint below 1000 cm^−1^ with that of the K_4_[α-SiW_12_O_40_] precursor.

Compound **1** was obtained in low yields as single crystals suitable for X-ray diffraction studies from the reaction of the [α-SiW_12_O_40_]^4−^ precursor and ferrocene (1:2 ratio) in a water/methanol mixture at reflux conditions. In contrast to what has been observed for the ferrocenium-POM salts reported to date, only partial oxidation of ferrocene took place in the formation of **1**, resulting in the first example in the literature in which ferrocenium (Fe^III^) and ferrocene (Fe^II^) species coexist in the same crystal structure. The formation of a compound combining Keggin-type POM clusters and ferrocenyl units such as **1** was firstly confirmed by FT-IR spectroscopy. Two parts can be clearly differentiated in the FT-IR spectrum of **1** (Figure 1): the inorganic fingerprint below 1000 cm^−1^ (a comparative detail with that of the K_4_[α-SiW_12_O_40_]·17H_2_O precursor is also depicted) and the organometallic region above. The spectrum of **1** exhibits the four characteristic bands of strong intensity (A, B, C and E) that unequivocally correspond to the plenary α-Keggin tungstosilicate anion [28] but with small red shifts of about 10 cm^−1^ that affect those three to which the ν_as_(W-O_t_) (O_t_: terminal O atom) vibrational mode contributes (bands A at 1011 cm^−1^, B at 972 cm^−1^ and C at 922 cm^−1^). The signal of medium-to-weak intensity that originates from the antisymmetric stretching vibration of the W-O-W bridges involving corner-sharing appears split at 880 and 856 cm^−1^ (signal D), whereas negligible modifications are noticed for those signals associated with the stretching vibrations of the W-O-W bridges between edge-sharing W centres (signal E at 785 cm^−1^) and the overall bending vibrations in both the oxometallic skeleton and the central heterogroup (signals F and G at 532 and 483 cm^−1^).

Focusing on the organic region, the presence of ferrocenyl species in **1** is confirmed by the signals associated with the stretching of the C(sp^2^)-H and C=C bonds that can be observed as peaks of medium intensity at ca. 3100 and 1410 cm^−1^, respectively. According to the literature, ferrocenyl groups in staggered (D_5d_) and eclipsed (D_5h_) conformation can be easily differentiated by IR spectroscopy because the fingerprint of both forms is substantially different in the 450–500 cm^−1^ region of the spectrum [29]. Unfortunately, the presence of the strong absorption bands of the [SiW_12_O_40_]^4−^ cluster below 1000 cm^−1^ and more specifically, of the broad signal F, corresponding to the (W-O_e_-W) + (Si-O_c_) combination (O_e_: bridging O atom between edge-sharing W centres; O_c_: central O atoms), makes impossible to perform such analysis in our case as it shadows this entire range.

### 2.2. Crystal Structure

**Figure 2:**
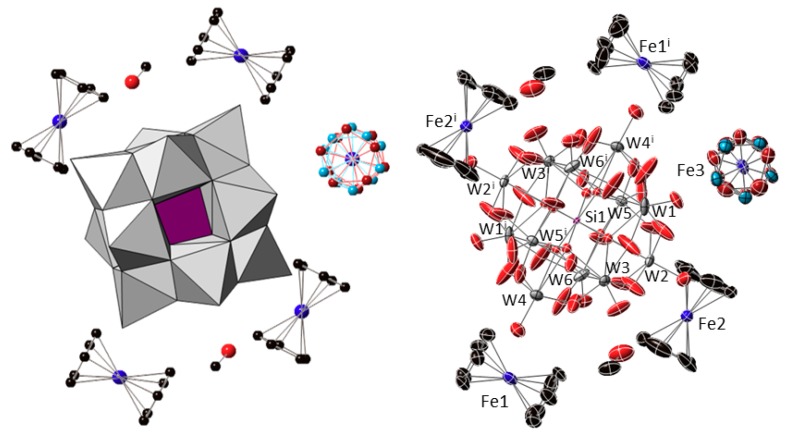
Combined polyhedral/ball-&-stick representation (**left**) and ORTEP view with 50% probability displacement ellipsoids (**right**) of the formula unit of **1**. Colour code: W and {WO_6_} octahedra, grey; Si and {SiO_4_} tetrahedron, purple; Fe, blue; C, black; O, red. The two positions over which the Cp ligands of the Fe3 unit are disordered are depicted in brown and light blue. Hydrogen atoms are omitted for clarity. Symmetry code: (i) 1 − *x*, −*y*, 1 − *z*.

Compound **1** crystallizes in the triclinic space group *P*-1 with the following content in the asymmetric unit: one half of the [α-SiW_12_O_40_]^4−^ cluster unit located on a centre of inversion; two ferrocenyl {Fe(C_5_H_5_)_2_} fragments placed in general positions (Fe1 and Fe2); one half of a centrosymmetric ferrocenyl {Fe(C_5_H_5_)_2_} unit, the cyclopentadienyl ligand of which is disordered over two positions that are related by an ideal in-plane rotation of 36° and show nearly equivalent population factors (Fe3); and one methanol molecule of crystallization (Figure 2). The [SiW_12_O_40_]^4−^ cluster shows the characteristic structure of the α-Keggin anion, which is constituted by four {W_3_O_13_} trimers composed each by three edge-sharing WO_6_ octahedra. These trimers are linked to each other and to the central {SiO_4_} tetrahedron through corner-sharing in ideal T_d_ symmetry. The central tetrahedron is disordered over two positions related by the centre of inversion on which the cluster is located, in such a way that a distorted {SiO_8_} cube with half-occupancies for the O sites is observed as a result. Table A1 (Appendix A) compiles the bond lengths (W–O and Si–O) and most relevant distances (W···W_trans_, W···Si and O···O_trans_) for the Keggin cluster in the title compound and their comparison with the magnitudes DFT-calculated for the [SiW_12_O_40_]^4−^ anion with idealized tetrahedral geometry [30]. In addition, Bond Valence Sum calculations [31] confirmed the highest oxidation state for all the tungsten atoms (W^VI^), indicating that no reduction took place for any of the POM metal centres. All the Fe–C bond lengths, Fe···Cg(Cp) distances and torsion angles between cyclopentadienyl rings for each crystallographically independent ferrocenyl unit in **1** are listed in Table 1. The presence of five ferrocenyl groups per [SiW_12_O_40_]^4−^ anion suggests different oxidation states for the iron centres belonging to the three crystallographically independent species that have been tentatively assigned as ferrocenium cations (Fe1 and Fe2) and ferrocene molecules (Fe3) in order to maintain the electroneutrality of the system. It is also worth mentioning that the two ferrocenium species in the organometallic sub-lattice exhibit different conformations: one of the cations (Fe1) displays an eclipsed configuration, whereas that of the second ferrocenium (Fe2) is staggered.

In order to crystallographically discriminate between ferrocenium cations and ferrocene molecules upon close inspection of the bond lengths and analyse the different geometrical conformations they can adopt, a topological study was carried out using all of the entries deposited in the CSD that contain isolated, pristine ferrocenyl species. Geometrical parameters of crystallographically independent 90 ferrocene and 49 ferrocenium fragments belonging to 119 different crystal structures have been determined in the CSD database (last visit: June 2018; last update: February 2018), excluding powder structures and those containing disordered fragments. All the Fe–C bond lengths, Cg(Cp)···Cg(Cp) and Fe···Cg(Cp) distances, torsion angles between cyclopentadienyl rings and oxidation states for the iron centres have been compiled in Table A2 (Appendix B).

**Figure 3:**
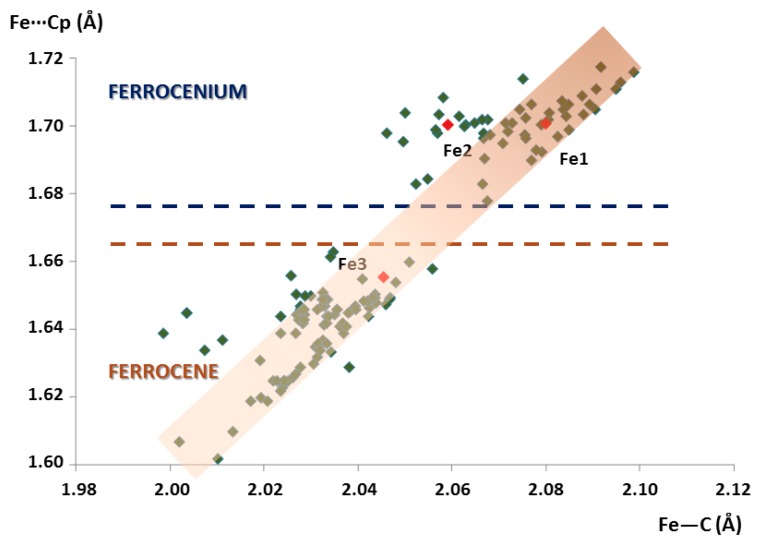
Plot of the average Fe···Cg(Cp) distances vs. Fe–C bond lengths for all the ferrocenyl moieties included in the search within the CSD database. Crystallographically independent ferrocenyl units in **1** are indicated in red.

The scatter plot of the average Fe···Cg(Cp) distances versus Fe–C bond lengths is depicted in Figure 3. It is worth mentioning that Fe···Cg(Cp) distances increase linearly with the Fe–C bond lengths, as defined by the <C–Fe···Cg(Cp)> angle of about 34–36° displayed by all the molecular units included in this search. All the ferrocenyl species can be graphically classified into two main groups depending on the oxidation state of the Fe atoms. The more stable Fe^II^ state for ferrocene moieties (obeys the 18-electron rule) exhibits shorter Fe–C and Fe···Cg(Cp) distances in the ca. 2.00–2.06 Å and 1.60–1.66 Å range respectively, whereas longer bond lengths in the ca. 2.05–2.11 Å and 1.68–1.72 Å range are found for ferrocenium cations. According to this distribution, the fact that the Fe···Cg(Cp) distances lay above or below 1.67 Å can be regarded as a direct method to unequivocally distinguish the nature of ferrocenyl units as ferrocene species or ferrocenium cations. When it comes to the crystal structure of **1**, two species belong to the latter group (Fe···Cg(Cp) = 1.70 Å) whereas the third is included in the former classification (Fe···Cg(Cp) = 1.66 Å). Therefore, we confirmed our initial assumption: Fe1 and Fe2 are ferrocenium cations, whereas Fe3 is a neutral ferrocene group.

**Figure 4:**
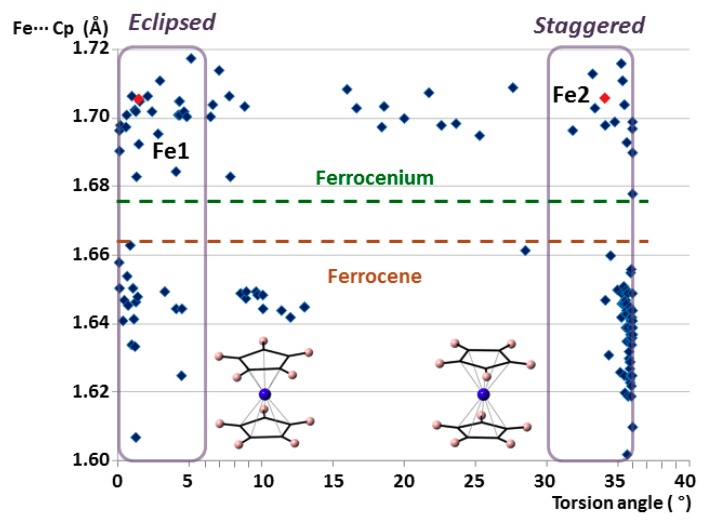
Plot of the average Fe···Cg(Cp) distances vs torsion angles for all the ferrocenyl moieties included in the search within the CSD database. The non-disordered crystallographically independent ferrocenyl units in **1** are indicated in red.

It is well known that ferrocenyl species can adopt two extreme conformations depending on the relative position of the cyclopentadienyl rings: eclipsed with ideal D_5h_ symmetry and staggered with ideal D_5d_ symmetry. Both configurations are very common because the rotation barrier along the C_5_ axis has been calculated to be as low as 0.9 ± 0.3 kcal mol^−1^ (≈4 kJ mol^−1^). [32] Analysis of the torsion angles between cyclopentadienyl rings determines the conformation of a given group that goes from 0° in a totally eclipsed form to 36° for a completely staggered configuration. Figure 4 displays the Fe···Cg(Cp) distance versus torsion-angle scatter plot for all the ferrocenyl units from the CSD database mentioned above. Considering an arbitrary criterion, torsion angles ranging from 0 to 6° have been classified as eclipsed, whereas those from 30 to 36° are staggered. The plot clearly shows that most of the species show extreme configurations, with less than 15% of the cases in intermediate states. In the case of ferrocenes, this effect is even more pronounced. More than 90% of the structures exhibit extreme conformations and 2/3 of the cases are staggered. Focusing on the non-disordered moieties in **1**, Fe1 is included within the group of eclipsed ferrocenium cations, whereas Fe2 is staggered.

**Figure 5:**
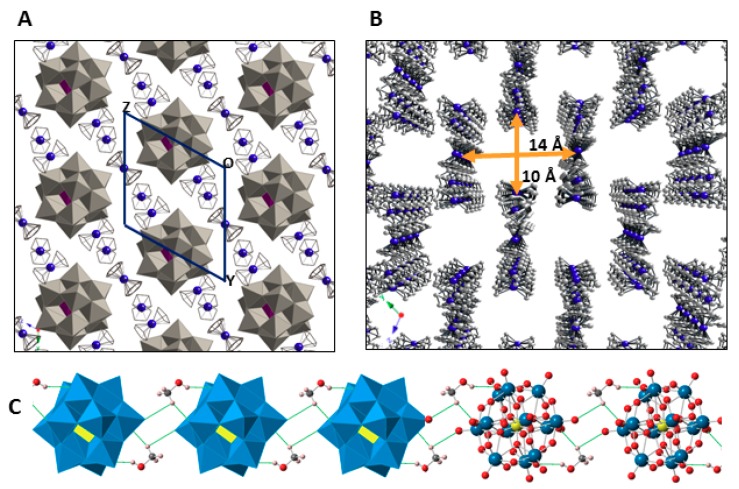
View of the crystal packing of **1** along the [100] direction (**A**). Channels formed by the organometallic sub-lattice. (**B**) Hydrogen atoms and methanol molecules are omitted for clarity. Hybrid polyhedral and ball & sticks representation of the hydrogen-bonded, methanol-bridged POM chain running along the [100] direction (**C**).

**Figure 6:**
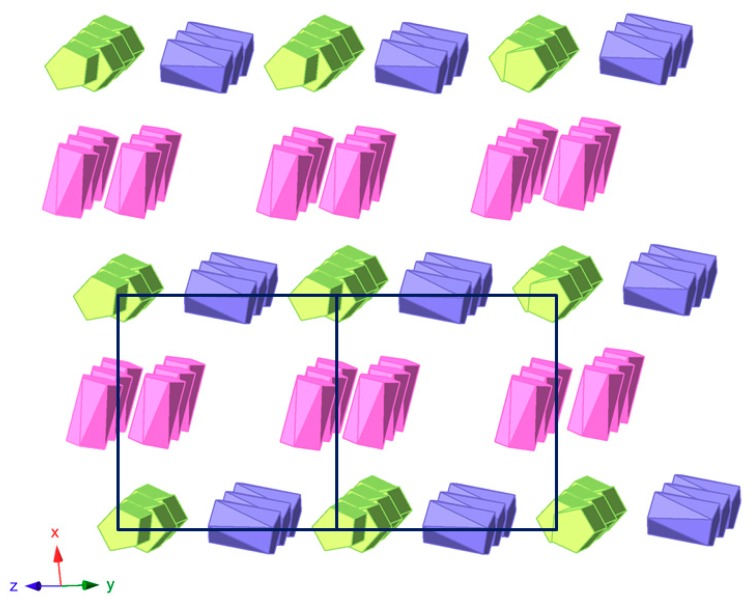
View of the organometallic sub-lattice formed by π-π interactions between ferrocenyl units in **1** along the [011] direction. Colour code: Fe1 ferrocenium (green), Fe2 ferrocenium (pink), Fe3 ferrocene (blue).

The crystal packing of **1** can be best described as an organometallic sub-lattice formed by ferrocenyl species that generate rectangular cavities of about 14 × 10 Å along the [100] direction where the [SiW_12_O_40_]^4−^ anions are hosted. Polyanions located in these cavities are linked to each other via weak O_POM_···C_MeOH_–O_MeOH_···O_POM_ type bonds involving methanol solvent molecules and surface O atoms from POM clusters (Figure 5). Pairs of Fe1 ferrocenium columns running along the [011] direction interact with each other in an anti-parallel manner through π-π stacking as can be viewed in Figure 6. The three dimensional network of the organometallic sub-lattice is completed by the other ferrocenyl units establishing weak T-type π-π interactions: Fe3 groups link Fe1 columns in the *yz* plane, whereas Fe2 units play a similar role along the crystallographic *x* axis. Geometrical parameters of the π-π interactions established between cyclopentadyenil rings are compiled in Table 2. Additionally, inorganic and organometallic components interact through C_Fc_–H···O_POM_ type contacts established between cyclopentadienyl rings from ferrocenyl groups and O atoms from the POM surface. Bond lengths and angles of such supramolecular interactions are summarized in Table 3.

### 2.3. Diffuse Reflectance UV-Vis Spectroscopy

**Figure 7:**
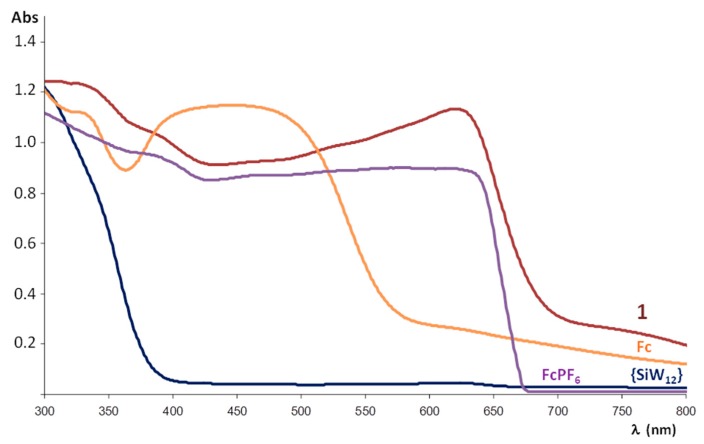
Diffuse Reflectance UV-Vis spectrum of powdered crystalline sample of **1** compared with those of the K_4_[SiW_12_O_40_]·17H_2_O precursor, ferrocene (Fc) and the FcPF_6_ salt.

To evaluate the electronic properties of the title compound, it has been analysed by diffuse reflectance UV-Vis spectroscopy. The spectra registered for a powdered crystalline sample of **1**, the K_4_[SiW_12_O_40_]·17H_2_O POM precursor, commercial ferrocene and the FcPF_6_ salt prepared for comparative purposes following reported procedures [33] are displayed in Figure 7. The electronic spectrum of the POM precursor shows a strong absorption band centred in the UV region (below 300 nm) that extends up to 375 nm and it is associated with the O→W ligand-to-metal charge transfer (LMCT) transition of the plenary inorganic framework. In the case of ferrocene, the band at ca. 340 and the broad adsorption in the blue region that extends from 360 to 580 nm (centred at ca. 450 nm) have been attributed to Fe (e_2g_) → Cp (e_1g_) charge transfer and symmetry-forbidden Fe (a_1g_) → Fe (e_1g_) transitions, respectively [34]. For its oxidized ferrocenium form in FcPF_6_, the continuous adsorption below 650 nm is in the origin of its dark blue colour. The O→W LMCT and adsorption bands belonging to both ferrocene and ferrocenium species can also be observed in the spectrum of **1**. However, the band at lower energy (ca. 640 nm) is exclusive for **1** and may be ascribed to an intermolecular charge-transfer transition between ferrocenyl donors and POM acceptors [19,21] since none of its constituents exhibit any absorption in this range.

### 2.4. ^57^Fe Mössbauer Spectroscopy

Preliminary Electronic Spin Resonance spectroscopy studies were conducted for **1** that proved how ferrocenium salts are silent at room temperature due to the short T1 relaxation time. The unpaired electron is not in the Fe^III^ centre and it seems to get delocalized all over the aromatic system. [35] Therefore, we decided to make use of ^57^Fe Mössbauer spectroscopy, because it allows for determining among others the oxidation and spin states of iron centres, as well as their symmetry, magnetic interactions and chemical environment. The technique is based on the absorption of energetically slightly different γ rays generated by Doppler effect in a radioactive source moving at speeds of several mm/s, in such a way that different absorption peaks are registered and their position is defined by the δ isomer shift. Nuclei in states with non-spherical charge distribution produce an asymmetrical quadrupolar electric field, which splits the nuclear energy levels. These are quantified by their quadrupolar splitting of the signals (Δ). Additionally, Zeeman splitting can be generated by magnetic coupling between centres. [36] 

**Figure 8:**
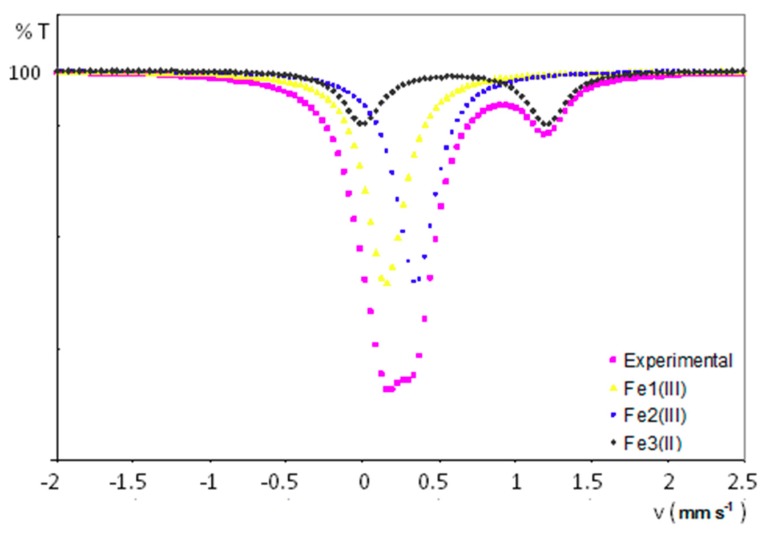
Experimental ^57^Fe Mössbauer spectrum and curve fits for each iron-containing species in **1**.

Figure 8 displays the experimental ^57^Fe Mössbauer spectrum for a powdered sample of **1**, together with the curve fits for each different iron-containing chemical species present in its crystal structure. All the experimental results are summarized in Table 4. The spectrum has been fitted to two singlets attributed to iron nuclei with a very similar chemical environment (δ = 0.27 and 0.44 mm/s) and a wide doublet with a quadrupolar splitting of 1.17 mm/s and a larger isomer shift of 0.71 mm/s. It is worth highlighting the absence of any extra peak, which indicates that paramagnetic centres are magnetically well isolated. The presence of both singlets compares well with the signals arising from ferrocenium units (Fe^III^ centres with spherical charge distribution) displaying isomer shifts that typically range from 0.30 to 0.65 mm/s. [37] Conversely, doublets with a chemical shift in the 0.50–1.0 range and quadrupolar splitting values of ca. 1–3 mm/s could be expected for ferrocene Fe^II^ nuclei. [38] These results are in line with the two crystallographically independent ferrocenium groups and the additional ferrocene molecule determined in the crystal structure of **1**. In fact, the relative atomic ratio for Fe1^III^:Fe2^III^:Fe3^II^ centres was calculated to be 2:2:1 from the integration of the area delimiting each of the sub-spectra, in good agreement with the molecular formula of **1**.

## 3. Experimental Section

### 3.1. Materials and Methods

The K_4_[α-SiW_12_O_40_]·17H_2_O precursor was synthesized following reported procedures [39] and identified by infrared spectroscopy (FT-IR). All other reagents were purchased from commercial sources and used without further purification. The FT-IR spectra were recorded as KBr pellets on a Shimadzu FTIR-8400S spectrophotometer (Shimadzu, Kyoto, Japan) in the 400−4000 cm^−1^ spectral range. The carbon and hydrogen contents were determined on a Perkin Elmer 2400 CHN analyser (PerkinElmer Inc., Waltham, MA, USA), whereas metal analyses (Fe) were performed on a Q-ICP-MS ThermoXSeries II analyser (Fisher Scientific International, Inc, Pittsburgh, PA, USA). Diffuse Reflectance studies were carried out on a UV-Vis-NIR Varian Cary 500 spectrophotometer (Varian, Palo Alto, CA, USA). The Mössbauer spectra were recorded at room temperature in transmission geometry using a conventional constant-acceleration spectrometer with a ^57^Co-Rh source calibrated with a Fe sheet (δ = −0.11 mm s^−1^). The fitting was performed using the NORMOS program (Universität Dortmund, Dortmund, Germany) [40].

### 3.2. Synthesis of [Fe(Cp)_2_]_4_[SiW_12_O_40_]·[Fe(Cp)_2_]·2CH_3_OH (1)

To a hot solution of K_4_[α-SiW_12_O_40_]·17H_2_O (990 mg, 0.30 mmol) in water (10 mL) Fe(Cp)_2_ (112 mg, 0.60 mmol) dissolved in methanol (5 mL) was added. The resulting yellow solution was refluxed for 2 h, stirred while cooling down to room temperature overnight and left to slowly evaporate in an open container. Purple prismatic crystals suitable for X-ray diffraction were obtained over a period of approximately two weeks (yield: 80 mg, 17% based on Fe). Elemental analysis (%) calc. for C_52_H_58_Fe_5_O_42_SiW_12_: C, 16.14; H, 1.51; Fe, 7.22. Found: C, 15.09; H, 1.42; Fe, 7.03. FT-IR (KBr, cm^−1^): 3101 (m), 1412 (m), 1011 (m), 972 (s), 922 (s), 88 0 (w), 856 (sh), 785 (s), 532 (m).

### 3.3. X-ray Crystallography

Crystallographic data for compound **1** are summarized in Table 5. Intensity data were collected at 100(2) K on an Oxford Diffraction Xcalibur (Rigaku Oxford Diffraction, Oxford, UK) single-crystal diffractometer (Mo Kα radiation, λ = 0.71073 Å) fitted with a Sapphire charge-coupled device detector. The data collection, unit cell determination, intensity data integration, routine corrections for Lorentz and polarization effects and analytical absorption correction with face indexing were processed using the CrysAlis software package (Rigaku Oxford Diffraction, Oxford, UK) [41]. The structure was solved using direct methods as implemented in SIR-2004 (Istituto di Cristallografia, CNR, Roma, Italy) [42] and refined by full-matrix least-squares analysis with the SHELXL-97 program (University of Göttingen, Göttingen, Germany) [43]. Heavy atoms (W, Fe, Si) were located in the initial resolution and the remaining light atoms (O, C) were located from successive Fourier maps. The C atoms from the ferrocene unit (Fe3) were disordered over two positions with 50% population factors. Thermal vibrations were treated anisotropically and those from non-disordered cyclopentadienyl C atoms were restrained to be similar to each other using default DELU commands. Thermal ellipsoids belonging to disordered cyclopentadienyl C atoms were restrained using more restrictive ISOR commands. For the All H atoms in the methanol molecules and cyclopentadienyl ligands were included in calculated positions and refined as riding atoms using default SHELXL parameters. Final geometrical calculations were carried out with PLATON (Utrecht University, Utrecht, The Netherlands) [44] as integrated in the WinGX (University of Glasgow, Glasgow, UK) crystallographic software package [45]. CCDC-1878742 (**1**) contains the supplementary crystallographic data for this paper. These data can be obtained free of charge from The Cambridge Crystallographic Data Centre via www.ccdc.cam.ac.uk/data_request/cif.

## 4. Conclusions

Compound [Fe^III^(Cp)_2_]_4_[SiW_12_O_40_]·[Fe^II^(Cp)_2_]·2CH_3_OH (**1**) constitutes the first example in the literature in which ferrocenium (Fe^III^) and ferrocene (Fe^II^) species coexist in the structure of a polyoxometalate-based salt. The asymmetric unit of **1** displays two crystallographically independent ferrocenium cations (one in an eclipsed *D_5h_* conformation and the other one in staggered *D_5d_*) and one half of a neutral ferrocene molecule disordered over two positions with similar population factors. The crystal packing of **1** can be best described as an organometallic sub-lattice of ferrocenyl-type species linked by a network of π-π interactions that generates rectangular cavities in which strings of Keggin anions and methanol molecules connected to each other via weak O_POM_···C_MeOH_–O_MeOH_···O_POM_ interactions, are hosted. The charge-transfer nature of the salt has been assessed by solid-state diffuse reflectance UV-Vis spectroscopy and ^57^Fe Mössbauer spectroscopy have proved to be a very useful tool to confirm the presence of magnetically isolated Fe^III^/Fe^II^ centres in a 4:1 ratio. Finally a thorough topological study on the pristine ferrocenyl species deposited in the CSD led us to conclude that (1) ferrocenyl groups tend to present extreme conformations; and (2) close inspection of geometrical parameters allows ferrocene neutral molecules and ferrocenium cations to be easily distinguished, because the later exhibit significant longer Fe···Cp distances (above or below 1.67 Å). For the near future, we plan to react transition metal- or lanthanide-containing POMs showing accessible centres with ferrocene derivatives with coordinating ability (e.g., ferrocene carboxylate, ferrocene-appended 2,2’-bipyrydine ligands) with the aim of studying the effect on their electronic properties (i.e., redox properties, charge-transfer processes).

## Figures and Tables

**Table 1:** Selected geometrical parameters (Å, °) for the three crystallographically independent ferrocenyl species in **1**.

	Fe1		Fe2		Fe3
C1	2.084 (9)	C11	2.044 (9)	C21A	2.077 (18)
C2	2.086 (8)	C12	2.059 (12)	C22A	2.048 (19)
C3	2.080 (7)	C13	2.057 (11)	C23A	2.02 (2)
C4	2.075 (7)	C14	2.043 (10)	C24A	2.037 (10)
C5	2.078 (8)	C15	2.034 (10)	C25A	2.019 (10)
C6	2.060 (9)	C16	2.062 (9)	C21B	1.98 (3)
C7	2.073 (8)	C17	2.084 (8)	C22B	2.03 (2)
C8	2.089 (10)	C18	2.083 (8)	C23B	2.09 (2)
C9	2.086 (10)	C19	2.062 (8)	C24B	2.095 (19)
C10	2.068 (9)	C20	2.049 (9)	C25B	2.03 (2)
Average	2.08	Average	2.06	Average	2.05
Fe···Cg(Cp1)	1.704	Fe···Cg(Cp3)	1.701	Fe···Cg(Cp5A)	1.648
Fe···Cg(Cp2)	1.699	Fe···Cg(Cp4)	1.708	Fe···Cg(Cp5B)	1.667
Torsion angle	0.94		34.53		-
Symmetry	D5_h_		D5_d_		Disordered

**Cg(Cpi)**: Centroid of the i cyclopentadienyl ring defined by the following atoms: i = 1: C1, C2, C3, C4, C5; i = 2: C6, C7, C8, C9, C10; i = 3: C11, C12, C13, C14, C15; i = 4: C16, C17, C18, C19, C20; i = 5: C21, C22, C23, C24, C25 (A and B represent the two equivalent positions in the disordered ferrocene unit).

**Table 2:** Geometrical parameters (Å, °) of the intermolecular π-π interactions in **1**.

π-π Interactions		Cg(Cp)··· Plane	ANG	Cg(Cp)··· Cg(Cp)	Slippage
Stacking	Cp1-Cp1^i^	3.445	0.00	3.497	0.603
	Cp2-Cp2^ii^	3.326	0.00	3.829	1.898
T-type	Cp1-Cp3^iii^	3.728	89.4 (5)	5.145	3.545
	Cp1-Cp5A	4.264	88.7 (9)	4.537	1.550
	Cp1-Cp5B	4.244	87.1 (11)	4.480	1.435
	Cp2-Cp4	4.680	88.3 (5)	4.720	0.618
	Cp3-Cp5A^iv^	4.938	88.4 (10)	5.079	1.188
	Cp3-Cp5B^iv^	4.873	87.4 (11)	4.989	1.061

**Cpi**: i cyclopentadienyl rings defined in Table 2. Cg(Cp)···plane: distance from one centroid to the plane containing the other ring. ANG: dihedral angle between planes containing both rings. Cg(Cp)··· Cg(Cp): distance between centroids. Slippage: distance between one centroid and its perpendicular projection to the plane containing the second ring. Symmetry codes: (i) −x, 1 − y, 1 − z; (ii) −x, −y, −z; (iii) −1 + x, y, z; (iv) 1 + x, y, z.

**Table 3:** Bond lengths (Å) and angles (°) for C–H···O type interactions in **1**.

D-A	D-H	H···A	D···A	D-H···A
*O1M–H1M···O13*	0.84	2.25	3.018 (12)	152
*C1M–H1M1···O14*	0.98	2.36	3.281 (15)	157
*C1M–H1M1···O4* ^i^	0.98	2.88	3.168 (16)	98
C4-H4···O4^i^	0.95	2.44	3.182 (12)	135
C7-H7···O5^ii^	0.95	2.55	3.450 (12)	157
C8-H8···O6^i^	0.95	2.48	3.286 (10)	143
C11-H11···O12^iii^	0.95	2.56	3.389 (13)	145
C12-H12···O2^iii^	0.95	2.48	3.225 (14)	135
C13-H13···O3^iv^	0.95	2.53	3.342 (14)	144
C13-H13···O8^iv^	0.95	2.51	3.371 (15)	150
C14-H14···O1	0.95	2.46	3.380 (14)	164
C15-H15···O6^v^	0.95	2.42	3.352 (16)	167
C16-H16···O6^iii^	0.95	2.44	3.240 (11)	142
C18-H18···O7^iv^	0.95	2.59	3.414 (15)	146
C19-H19···O1^iv^	0.95	2.59	3.308 (11)	133
C20-H20···O15	0.95	2.56	3.272 (11)	132
C24B-*H*24B···O16i^iii^	0.95	2.59	3.427 (12)	147

**D** = donor; **A** = acceptor. Symmetry codes: (i) 1 − x, −y, 1 − z; (ii) −1 + x, y, −1 + z; (iii) x, y, −1 + z; (iv) 1 − x, 1 − y, 1 − z; (v) 1 − x, −y, 1 − z.

**Table 4:** Experimental results obtained from the ^57^Fe Mössbauer spectrum of **1**.

Signal	δ (mm/s)	Multiplicity	Δ (mm/s)	Oxidation State	Area	Atomic %
Fe1	0.27	1	-	III	2	40
Fe2	0.44	1	-	III	2	40
Fe3	0.71	2	1.17	II	1	20

δ isomer shifts are relative to the α-Fe.

**Table 5:** Crystallographic data for **1**.

Parameters	1
Formula	C_52_H_58_Fe_5_O_42_SiW_12_
FW (g mol^−1^)	3868.40
Crystal System	Triclinic
Space Group	*P*–1
*a* (Å)	12.5120 (5)
*b*(Å)	13.0831 (6)
*c* (Å)	13.3076 (6)
α (°)	117.296 (5)
β (°)	95.632 (3)
γ (°)	101.909 (4)
*V* (Å^3^)	1874.03 (18)
*Z*	1
*ρ*_calcd_ (g cm^−3^)	3.478
*μ* (mm^−1^)	19.651
Collected Reflections	19620
Unique Reflections (*R_int_*)	8904 (0.036)
Observed Reflections [*I* > 2*σ*(*I*)]	6061
Parameters	505
Restrains	120
*R*(*F*) ^a^ [*I* > 2*σ*(*I*)]	0.036
*wR*(*F*^2^) ^a^ [all data]	0.072
GoF	0.860

^a^*R*(*F*) = Σ||*F*_o_ − *F*_c_||/Σ|*F*_o_|; *wR*(*F*^2^) = {Σ[*w*(*F*_o_^2^ − *F*_c_^2^)^2^]/Σ[*w*(*F*_o_^2^)^2^]}^1/2^.

## Supplementary Materials

The following are available online: CIF file for the crystal structure of **1** reported in this paper.

## Appendix A

**Table A1:** W-O and Si-O_C_ bond lengths and W···Si, W···W_trans_ and O···O_trans_ (Å) distances for the inorganic building block [SiW_12_O_40_]^4−^ in **1** and their comparison with those of the optimized polyanion.

**1**												
		**W1**		**W2**		**W3**		**W4**		**W5**		**W6**
*O_t_*	**O1**	1.670 (5)	**O2**	1.655 (6)	**O3**	1.667 (6)	**O4**	1.658 (6)	**O5**	1.669 (7)	**O6**	1.666 (6)
*O_b_*	**O7**	1.872 (8)	**O12**	1.891 (6)	**O14**	1.870 (6)	**O16**	1.885 (8)	**O16**	1.894 (6)	**O17**	1.885 (7)
	**O10**	1.880 (8)	**O7**	1.904 (7)	**O8**	1.885 (8)	**O11**	1.892 (7)	**O15**	1.893 (6)	**O13**	1.891 (7)
	**O11**	1.887 (8)	**O13**	1.909 (6)	**O9**	1.891 (6)	**O18**	1.895 (8)	**O17**	1.900 (6)	**O10**	1.899 (7)
	**O9**	1.888 (7)	**O8**	1.913 (8)	**O15**	1.900 (7)	**O14**	1.896 (7)	**O12**	1.905 (6)	**O18**	1.900 (8)
*O_c_*	**O22**	2.372 (9)	**O19**	2.367 (11)	**O19**	2.344 (10)	**O22**	2.323 (10)	**O20**	2.348 (10)	**O22**	2.360 (11)
**[SiW_12_O_40_]^4−^**		**1**			**Optimized [30]**							
		*Range*		*Average*								
*W–O_c_*		2.323–2.461		2.385	2.325							
*W–O_b_*		1.870–1.913		1.893	1.916							
*W–O_t_*		1.655–1.670		1.664	1.743							
*Si–O_c_*		1.591–1.705		1.644	1.667							
*W···Si*		3.516–3.541		3.529	3.588							
*W···W_trans_*		7.032–7.082		7.057	7.172							
*O···O_trans_*		10.364–10.421		10.386	10.619							

*O_c_*: central oxygen atom; *O_b_*: bridging oxygen atoms between W centres; *O_t_*: terminal oxygen atoms.

## Appendix B

**Table A2:** Selected distances (Å), torsion angles (T, °) and oxidation states of Fe centres (OS) for all the structures containing pristine ferrocenyl units included in the CSD database.

Refcode	Fe–C										*Mean*	Cg···Cg′	Fe···Cg		T	OS
AFALAV	2.074	2.076	2.071	2.065	2.071	2.075	2.086	2.088	2.077	2.071	2.075	3.395	1.697	1.698	18.844	III
AFALID	2.098	2.107	2.081	2.081	2.107	2.081	2.081	2.107	2.098	2.107	2.095	3.422	1.711	1.711	35.251	III
AFALID01	2.081	2.093	2.068	2.068	2.093	2.068	2.068	2.093	2.081	2.093	2.081	3.408	1.704	1.704	35.423	III
AFALOJ	2.086	2.074	2.072	2.094	2.076	2.078	2.079	2.081	2.098	2.104	2.084	3.406	1.704	1.702	17.247	III
AGEFEX	2.06	2.027	2.039	2.056	2.046	2.066	2.051	2.03	2.077	2.047	2.050	3.407	1.702	1.706	5.975	III
BEJLOR (Fe1)	2.093	2.065	2.028	2.009	2.038	2.058	2.003	2.043	2.038	2.062	2.044	3.293	1.650	1.646	1.615	II
BEJLOR (Fe2)	2.038	2.051	2.064	2.065	2.049	1.986	2.078	2.087	2.058	2.003	2.048	3.305	1.658	1.650	0.369	II
COTPUW02	2.056	2.046	2.103	2.046	2.056	2.07	2.073	2.087	2.073	2.07	2.068	3.394	1.698	1.697	0	III
DOMZEM	2.12	2.115	2.085	2.066	2.08	2.064	2.074	2.087	2.111	2.102	2.090	3.41	1.707	1.703	3.723	III
DOQHOG	1.985	1.991	1.959	1.934	2.01	2.01	1.934	1.959	1.991	1.985	1.976	3.118	1.559	1.559	0	II
FANNUE	2.01	2.01	2.015	2.01	2.01	2.015	2.01	2.01	2.01	2.01	2.011	3.275	1.637	1.637	34.704	II
FANNUE01	2.001	2.005	2.054	2.005	2.001	2.054	2.005	2.001	2.001	2.005	2.013	3.221	1.610	1.610	34.268	II
FEHYAS02	2.029	2.044	2.049	2.037	2.033	2.025	2.042	2.024	2.019	2.024	2.033	3.283	1.644	1.639	0.241	II
FEHYAS03	2.04	2.047	2.044	2.037	2.045	2.036	2.043	2.037	2.03	2.024	2.038	3.291	1.647	1.644	0.622	II
FEHYAS04	1.995	2.025	2.047	2.024	2.01	2.02	1.999	2.017	1.99	1.965	2.007	3.263	1.643	1.625	1.051	II
FEOCAS	2.074	2.062	2.063	2.052	2.065	2.066	2.064	2.068	2.078	2.083	2.067	3.403	1.699	1.705	2.783	III
FERCBI10	2.068	2.073	2.073	2.114	2.086	2.065	2.09	2.071	2.054	2.062	2.076	3.404	1.706	1.699	0.487	III
FERCBI11	2.109	2.086	2.063	2.073	2.105	2.102	2.094	2.068	2.085	2.107	2.089	3.412	1.705	1.708	2.391	III
FEROCE16 (Fe1)	2.043	2.038	2.046	2.051	2.049	2.05	2.045	2.048	2.048	2.048	2.047	3.297	1.648	1.650	8.735	II
FEROCE16 (Fe2)	2.041	2.045	2.053	2.048	2.046	2.046	2.048	2.045	2.042	2.044	2.046	3.295	1.649	1.646	9.023	II
FEROCE17 (Fe1)	2.038	2.035	2.041	2.048	2.044	2.049	2.041	2.048	2.044	2.047	2.043	3.299	1.648	1.651	9.118	II
FEROCE17 (Fe2)	2.035	2.043	2.05	2.046	2.04	2.042	2.048	2.042	2.038	2.045	2.043	3.298	1.65	1.649	9.512	II
FEROCE18 (Fe1)	2.034	2.035	2.039	2.043	2.045	2.05	2.043	2.046	2.041	2.043	2.042	3.297	1.647	1.650	9.837	II
FEROCE18 (Fe2)	2.027	2.039	2.052	2.045	2.036	2.043	2.046	2.043	2.034	2.046	2.041	3.296	1.648	1.649	10.283	II
FEROCE24	2.055	2.051	2.063	2.063	2.051	2.054	2.052	2.052	2.054	2.062	2.056	3.316	1.655	1.661	0.069	II
FEROCE26	2.012	2.035	2.042	2.023	2.005	2.023	2.005	2.012	2.035	2.042	2.023	3.287	1.644	1.644	35.998	II
FEROCE27	2.009	2.012	2.031	2.039	2.026	2.031	2.039	2.026	2.009	2.012	2.023	3.277	1.639	1.639	35.983	II
FEROCE35	2.031	2.01	2.005	2.017	2.022	2.017	2.022	2.031	2.01	2.005	2.017	3.238	1.619	1.619	35.735	II
FEROCE36	2.038	2.011	2.007	2.016	2.024	2.016	2.024	2.038	2.011	2.007	2.019	3.239	1.620	1.620	35.44	II
FEROCE37	2.041	2.016	2.009	2.018	2.033	2.018	2.033	2.041	2.016	2.009	2.023	3.244	1.622	1.622	35.585	II
FEROCE38	2.032	2.012	2.008	2.017	2.034	2.017	2.034	2.032	2.012	2.008	2.021	3.238	1.619	1.619	35.778	II
FEROCE39	2.041	2.011	2.016	2.016	2.035	2.016	2.035	2.041	2.011	2.016	2.024	3.247	1.623	1.623	35.762	II
FEROCE40	2.036	2.015	2.016	2.021	2.033	2.021	2.033	2.036	2.015	2.016	2.024	3.249	1.625	1.625	35.833	II
FEROCE41	2.037	2.018	2.014	2.021	2.035	2.021	2.035	2.037	2.018	2.014	2.025	3.251	1.625	1.625	35.731	II
FEROCE42	2.026	2.052	2.021	2.012	2.019	2.021	2.052	2.026	2.019	2.012	2.026	3.252	1.626	1.626	34.943	II
FEROCE43	2.031	2.017	2.017	2.02	2.035	2.02	2.035	2.031	2.017	2.017	2.024	3.247	1.624	1.624	35.825	II
FEROCE44	2.04	2.022	2.018	2.022	2.036	2.022	2.036	2.04	2.022	2.018	2.028	3.258	1.629	1.629	35.816	II
FEROCE45	2.044	2.021	2.018	2.028	2.041	2.028	2.041	2.044	2.021	2.018	2.030	3.26	1.630	1.630	35.881	II
FEROCE46	2.044	2.021	2.02	2.026	2.045	2.026	2.045	2.044	2.021	2.02	2.031	3.264	1.632	1.632	35.498	II
FEROCE47	2.045	2.025	2.024	2.027	2.036	2.027	2.036	2.045	2.025	2.024	2.031	3.272	1.636	1.636	35.870	II
FEROCE48	2.044	2.024	2.029	2.032	2.037	2.032	2.037	2.044	2.024	2.029	2.033	3.271	1.636	1.636	35.723	II
FEROCE49	2.040	2.033	2.027	2.02	2.034	2.020	2.034	2.04	2.033	2.027	2.031	3.270	1.635	1.635	35.471	II
FEROCE50	2.053	2.028	2.036	2.023	2.044	2.023	2.044	2.053	2.028	2.036	2.037	3.277	1.639	1.639	35.602	II
FEROCE51	2.054	2.026	2.026	2.03	2.051	2.026	2.03	2.051	2.054	2.026	2.037	3.282	1.641	1.641	35.753	II
FEROCE52	2.045	2.029	2.028	2.032	2.049	2.032	2.049	2.045	2.029	2.028	2.037	3.285	1.642	1.642	35.794	II
FEROCE53	2.052	2.024	2.027	2.031	2.046	2.027	2.031	2.046	2.052	2.024	2.036	3.282	1.641	1.641	35.743	II
FEROCE54	2.051	2.028	2.022	2.025	2.042	2.025	2.042	2.051	2.028	2.022	2.034	3.288	1.644	1.644	35.784	II
FEROCE55	2.073	2.016	2.017	2.011	2.06	2.017	2.011	2.06	2.073	2.016	2.035	3.292	1.646	1.646	34.983	II
FEROCE56	2.061	2.025	2.016	2.018	2.036	2.016	2.018	2.036	2.061	2.025	2.031	3.293	1.646	1.646	34.804	II
FEROCE57	2.062	2.018	2.019	2.015	2.028	2.019	2.015	2.028	2.062	2.018	2.028	3.292	1.646	1.646	34.976	II
FEROCE58	2.060	2.022	2.021	2.013	2.022	2.021	2.013	2.022	2.06	2.022	2.028	3.294	1.647	1.647	34.540	II
FEROCE59	2.049	2.04	2.009	2.030	2.034	2.009	2.030	2.034	2.049	2.040	2.032	3.301	1.651	1.651	35.456	II
FEROCE60	2.059	1.981	2.009	2.033	2.085	2.009	1.981	2.059	2.085	2.033	2.033	3.299	1.649	1.649	35.317	II
FEROCE61	2.011	2.072	2.072	1.970	2.036	1.970	2.036	2.011	2.072	2.072	2.032	3.297	1.649	1.649	35.955	II
FEROCE62	2.024	2.017	1.982	2.067	2.073	1.982	2.017	2.024	2.073	2.067	2.033	3.297	1.649	1.649	35.283	II
FEROCE63	2.030	2.038	1.995	2.027	2.052	1.995	2.038	2.030	2.052	2.027	2.028	3.287	1.643	1.643	35.361	II
FEROCE64	2.032	2.042	2.025	2.026	2.039	2.025	2.042	2.032	2.039	2.026	2.033	3.294	1.647	1.647	35.406	II
FEROCE65	2.031	2.052	2.044	2.046	2.024	2.046	2.024	2.031	2.052	2.044	2.039	3.292	1.646	1.646	35.621	II
FEROCE66	2.031	2.026	2.001	2.042	2.033	2.001	2.026	2.031	2.033	2.042	2.027	3.279	1.639	1.639	35.444	II
FEROCE67	2.055	2.018	2.011	2.016	2.028	2.011	2.016	2.028	2.055	2.018	2.026	3.312	1.656	1.656	34.620	II
FEROCE68	2.065	2.023	2.014	2.022	2.037	2.014	2.022	2.037	2.065	2.023	2.032	3.293	1.647	1.647	34.804	II
FEROCE69	2.045	2.030	2.011	2.028	2.029	2.011	2.028	2.029	2.045	2.03	2.029	3.300	1.650	1.650	35.185	II
FEROCE70	2.044	2.019	2.035	2.017	2.047	2.035	2.019	2.044	2.047	2.017	2.032	3.299	1.649	1.649	35.774	II
FEROCE71	2.047	2.032	2.019	2.028	2.023	2.019	2.028	2.023	2.047	2.032	2.030	3.300	1.650	1.650	35.510	II
FEROCE72	2.044	2.024	2.015	2.019	2.039	2.015	2.019	2.039	2.044	2.024	2.028	3.284	1.642	1.642	34.859	II
FEROCE73	2.036	2.026	2.019	2.029	2.028	2.019	2.029	2.028	2.036	2.026	2.028	3.291	1.646	1.646	35.373	II
FEROCE74	2.044	2.031	2.024	2.03	2.037	2.024	2.03	2.037	2.044	2.031	2.033	3.298	1.649	1.649	35.724	II
FEROCE75	2.051	2.02	2.025	2.025	2.045	2.025	2.025	2.045	2.051	2.02	2.033	3.287	1.644	1.644	34.980	II
FEROCE76	2.044	2.026	2.02	2.028	2.048	2.02	2.028	2.048	2.044	2.026	2.033	3.285	1.642	1.642	35.101	II
FEROCE77	2.053	2.03	2.026	2.028	2.052	2.026	2.028	2.052	2.053	2.030	2.038	3.289	1.645	1.645	35.651	II
FEROCE78	2.05	2.026	2.023	2.026	2.037	2.026	2.037	2.05	2.026	2.023	2.032	3.274	1.637	1.637	35.912	II
FEROCE79	2.057	2.026	2.015	2.033	2.059	2.033	2.059	2.057	2.026	2.015	2.038	3.257	1.629	1.629	34.954	II
FEROCE80	2.038	2.045	2.028	2.02	2.027	2.028	2.045	2.038	2.027	2.02	2.032	3.269	1.635	1.635	35.770	II
FEROCE81	2.043	2.045	2.029	2.021	2.021	2.029	2.045	2.043	2.021	2.021	2.032	3.268	1.634	1.634	35.826	II
FEROCE82	2.043	2.014	2.009	2.027	2.027	2.027	2.027	2.043	2.014	2.009	2.024	3.249	1.625	1.625	35.719	II
FEROCE83	2.046	2.014	2.012	2.023	2.038	2.023	2.038	2.046	2.014	2.012	2.027	3.254	1.627	1.627	35.783	II
FEROCE84	2.04	2.007	2.006	2.022	2.038	2.022	2.038	2.04	2.007	2.006	2.023	3.250	1.625	1.625	35.797	II
FEROCE85	2.063	2.026	1.948	2.04	2.059	1.948	2.026	2.063	2.059	2.04	2.027	3.286	1.643	1.643	35.437	II
FERRIC01	2.06	2.06	2.063	2.06	2.063	2.064	2.07	2.064	2.081	2.081	2.067	3.395	1.701	1.695	0.507	III
FUZGOY	2.086	2.075	2.054	2.097	2.074	2.055	2.064	2.068	2.063	2.071	2.071	3.390	1.695	1.695	25.674	III
GOFLUI	2.093	1.95	1.98	1.975	1.941	1.955	2.035	2.001	2.028	1.94	1.999	3.135	1.564	1.573	4.696	II
GOKRIH	2.057	2.062	2.116	2.047	2.05	2.05	2.047	2.116	2.062	2.057	2.066	3.365	1.683	1.683	7.076	III
GUNGAX	2.068	2.064	2.075	2.094	2.08	2.08	2.072	2.033	2.023	2.074	2.066	3.403	1.708	1.696	4.352	III
GUNGEB	2.067	2.068	2.056	2.087	2.067	2.006	2.035	2.054	2.086	2.042	2.057	3.395	1.700	1.696	22.686	III
GUNGIF	2.074	2.064	2.057	2.037	2.05	2.057	2.037	2.05	2.074	2.064	2.056	3.397	1.699	1.699	32.324	III
HARGIQ	2.018	2.027	2.037	2.043	2.022	2.034	2.024	2.027	2.029	2.024	2.028	3.289	1.645	1.644	4.206	II
HIGHUA	2.082	2.092	2.079	2.08	2.069	2.08	2.079	2.092	2.082	2.069	2.080	3.404	1.702	1.702	0.333	III
HUZLAR	2.055	2.06	2.054	2.038	2.042	2.048	2.054	2.023	2.054	2.01	2.044	3.288	1.658	1.631	6.107	II
IMUBEZ	1.999	2.005	2.008	1.994	2.011	1.999	2.011	1.994	2.008	2.005	2.003	3.291	1.645	1.645	13.012	II
INIKIZ (Fe1)	2.069	2.05	2.085	2.074	2.029	2.074	2.029	2.069	2.05	2.085	2.061	3.405	1.703	1.703	35.837	III
INIKIZ (Fe2)	2.041	2.028	2.016	2.027	2.073	2.04	2.052	2.049	2.03	1.984	2.034	3.323	1.661	1.662	24.793	II
INIKIZ (Fe3)	2.085	2.066	2.078	2.077	2.083	2.078	2.077	2.083	2.085	2.066	2.078	3.387	1.693	1.693	35.733	III
INIKIZ (Fe4)	2.077	2.076	2.092	2.051	2.046	2.097	2.09	2.056	2.107	2.058	2.075	3.427	1.708	1.720	9.735	III
INIKIZ (Fe5)	2.007	2.027	2.036	2.035	1.99	2.036	2.027	2.007	1.99	2.035	2.019	3.262	1.631	1.631	35.683	II
INIKIZ(Fe6)	2.098	2.095	2.086	2.054	2.057	2.089	2.081	2.049	2.056	2.091	2.076	3.392	1.703	1.690	28.649	III
IVUHIQ (Fe1)	2.031	2.042	2.034	2.026	2.024	2.04	2.041	2.039	2.035	2.037	2.035	3.288	1.641	1.648	9.552	II
IVUHIQ (Fe2)	2.014	2.028	2.028	2.022	2.029	2.037	2.035	2.019	2.03	2.038	2.028	3.284	1.638	1.646	12.375	II
JAHQAK	2.038	2.057	2.069	2.066	2.051	2.058	2.078	2.06	2.046	2.048	2.057	3.407	1.705	1.702	16.247	III
JALWIC (Fe1)	2.051	2.042	2.065	2.077	2.042	2.113	2.072	2.018	2.078	2.07	2.063	3.400	1.707	1.694	4.775	III
JALWIC (Fe2)	2.095	2.073	2.044	2.081	2.082	2.07	2.071	2.073	2.085	2.069	2.074	3.410	1.706	1.704	2.117	III
JUXZEJ	2.074	2.065	2.011	2.024	2.076	2.026	2.043	2.05	2.056	2.07	2.049	3.39	1.693	1.698	1.978	III
KAFMAG	2.039	2.013	2.015	2.045	2.063	2.071	2.048	2.046	2.027	2.054	2.042	3.287	1.637	1.651	11.147	II
KALGEL	2.045	2.033	2.000	2.063	2.032	2.063	2.032	2.045	2.033	2.000	2.035	3.327	1.663	1.663	0.239	II
KEFXUO (Fe1)	2.06	2.079	2.045	2.038	2.032	2.038	2.032	2.06	2.079	2.045	2.051	3.319	1.660	1.660	35.92	II
KEFXUO (Fe3)	2.053	2.034	2.091	2.013	2.039	2.013	2.039	2.053	2.034	2.091	2.046	3.397	1.698	1.698	32.032	III
KEFXUO03 (Fe1)	2.086	2.101	2.095	2.074	2.068	2.074	2.068	2.086	2.101	2.095	2.085	3.399	1.699	1.699	36.000	III
KEFXUO03 (Fe2)	2.09	2.064	2.042	2.055	2.086	2.055	2.086	2.09	2.064	2.042	2.067	3.356	1.678	1.678	35.987	III
KOVMUD	2.046	2.037	2.063	2.063	2.037	2.061	2.051	2.051	2.061	2.052	2.052	3.366	1.694	1.672	0.069	III
KUVNOE	2.046	2.041	2.041	2.044	2.047	2.049	2.039	2.051	2.058	2.051	2.047	3.299	1.648	1.651	2.706	II
KUVNOE01	2.049	2.029	2.04	2.044	2.048	2.053	2.039	2.038	2.043	2.052	2.043	3.301	1.649	1.652	1.307	II
LAWTIM	2.093	2.092	2.092	2.11	2.092	2.11	2.092	2.093	2.092	2.092	2.096	3.425	1.713	1.713	33.351	III
LETSAF	2.082	2.077	2.065	2.067	2.076	2.07	2.053	2.071	2.072	2.08	2.071	3.402	1.700	1.702	4.472	III
LETSAF01 (Fe1)	2.114	2.091	2.116	2.091	2.073	2.06	2.08	2.095	2.091	2.095	2.091	3.422	1.715	1.707	3.588	III
LETSAF01 (Fe2)	2.068	2.098	2.116	2.059	2.073	2.097	2.093	2.078	2.08	2.117	2.088	3.407	1.702	1.705	7.904	III
LIYNAI (Fe1)	2.076	2.062	2.067	2.072	2.022	2.062	2.072	2.081	2.056	2.055	2.062	3.397	1.699	1.701	21.828	III
LIYNAI (Fe2)	2.062	2.054	2.078	2.083	2.057	2.074	2.095	2.09	2.073	2.061	2.073	3.401	1.697	1.705	3.528	III
LONGOK	2.039	2.038	2.044	2.046	2.044	2.039	2.045	2.05	2.037	2.041	2.042	3.293	1.646	1.647	1.214	II
LUZJIB	2.024	2.03	2.015	2.024	2.024	2.017	2.027	2.032	2.053	2.022	2.027	3.288	1.646	1.643	4.092	II
MACWUJ	2.046	2.1	2.115	2.088	2.045	2.057	2.086	2.097	2.077	2.057	2.077	3.412	1.708	1.705	8.353	III
NAHMEP	2.036	2.04	2.035	2.031	2.035	2.035	2.031	2.035	2.036	2.04	2.035	3.292	1.646	1.646	35.310	II
NIRSAK	2.082	2.085	2.087	2.064	2.08	2.074	2.109	2.109	2.083	2.06	2.083	3.415	1.711	1.704	19.850	III
NOHKAX	2.054	2.032	2.037	2.034	2.047	2.034	2.047	2.054	2.032	2.037	2.041	3.309	1.655	1.655	35.656	II
NUMXAU	2.068	2.068	2.093	2.09	2.093	2.047	2.045	2.047	2.048	2.048	2.065	3.402	1.710	1.692	0.141	III
OCUJIF	2.02	2.006	1.996	1.974	1.996	1.974	1.996	2.02	2.006	1.996	1.998	3.279	1.639	1.639	33.580	II
POTTOH (Fe1)	2.079	2.093	2.087	2.08	2.073	2.083	2.091	2.091	2.085	2.074	2.084	3.41	1.705	1.705	4.606	III
POTTOH (Fe2)	2.039	2.036	2.035	2.035	2.033	2.035	2.035	2.042	2.041	2.031	2.036	3.282	1.640	1.642	0.702	II
POTTOH (Fe3)	2.079	2.076	2.082	2.08	2.087	2.08	2.071	2.07	2.081	2.083	2.079	3.401	1.702	1.699	4.256	III
PUVFIV	2.06	2.06	2.061	2.067	2.061	2.064	2.069	2.064	2.08	2.08	2.067	3.393	1.698	1.695	0.166	III
QIDREB	2.044	2.037	2.026	2.032	2.039	2.043	2.044	2.043	2.045	2.042	2.039	3.290	1.637	1.653	8.297	II
RAMTII (Fe1)	2.053	2.075	2.108	2.105	2.071	2.108	2.105	2.071	2.053	2.075	2.082	3.393	1.697	1.697	35.970	III
RAMTII (Fe2)	2.06	2.071	2.09	2.091	2.072	2.09	2.071	2.06	2.072	2.091	2.077	3.379	1.690	1.690	35.978	III
RETMOS (Fe1)	2.101	2.111	2.071	2.091	2.119	2.091	2.119	2.101	2.111	2.071	2.099	3.432	1.716	1.716	35.505	III
RETMOS (Fe2)	2.069	2.096	2.035	2.053	2.082	2.087	2.074	2.076	2.063	2.083	2.072	3.397	1.704	1.693	19.144	III
TIBCUE	1.872	2.019	2.044	2.087	2.028	2.044	2.087	2.028	1.872	2.019	2.010	3.205	1.602	1.602	32.946	II
VOVLOJ	2.04	2.042	2.045	2.035	2.03	2.034	2.041	2.045	2.049	2.032	2.039	3.294	1.647	1.647	0.174	II
XITDIP	2.109	2.089	2.081	2.088	2.08	2.079	2.088	2.091	2.102	2.069	2.088	3.417	1.711	1.707	25.129	III
YIPQIX	2.06	2.06	2.066	2.059	2.066	2.065	2.071	2.065	2.078	2.078	2.067	3.380	1.692	1.689	0.308	III
ZAGXEK	2.087	2.062	2.066	2.064	2.046	2.055	2.063	2.058	2.055	2.024	2.058	3.415	1.709	1.708	18.764	III
ZOZLEF	2.097	2.08	2.067	2.076	2.094	2.098	2.085	2.073	2.078	2.099	2.085	3.412	1.707	1.706	0.892	III

Cg = ring centroid; T = torsion angle between the two cyclopentadienyl rings.
