# Supplementary material for: A Charge-Transfer Salt Based on Ferrocene/Ferrocenium Pairs and Keggin-Type Polyoxometalates
Source: Molecules. 2018 Nov 30;23(12):3150. doi: 10.3390/molecules23123150 (PMC6321299; doi:10.3390/molecules23123150)

checkCIF/PLATON report

Structure factors have been supplied for datablock(s) am81_100

THIS REPORT IS FOR GUIDANCE ONLY. IF USED AS PART OF A REVIEW PROCEDURE FOR PUBLICATION, IT SHOULD NOT REPLACE THE EXPERTISE OF AN EXPERIENCED CRYSTALLOGRAPHIC REFEREE.

No syntax errors found. CIF dictionary Interpreting this report

Datablock: am81_100

```
Bond precision:  C-C = 0.0137 A          Wavelength=0.71073

Cell:           a=12.5120(5)       b=13.0831(6)     c=13.3076(6)
                alpha=117.296(5)   beta=95.632(3)   gamma=101.909(4)
Temperature:    100 K
```

	Calculated	Reported
Volume	1847.03(18)	1847.03(18)
Space group	P -1	P -1
Hall group	-P 1	-P 1
Moiety formula	O40 Si W12, 5(C10 H10 Fe), 2(C H4 O)	Si W12 O40, 5(C10 H10 Fe), 2(C H4 O)
Sum formula	C52 H58 Fe5 O42 Si W12	C52 H58 Fe5 O42 Si W12
Mr	3868.40	3868.40
Dx,g cm-3	3.478	3.478
Z	1	1
Mu (mm-1)	19.651	19.651
F000	1738.0	1738.0
F000’	1731.74	
h,k,lmax	16,17,17	16,17,17
Nref	8922	8904
Tmin,Tmax	0.248,0.608	0.230,0.621
Tmin’	0.034	

```
Correction method= # Reported T Limits: Tmin=0.230 Tmax=0.621
AbsCorr = ANALYTICAL

Data completeness= 0.998          Theta(max)= 28.000

R(reflections)= 0.0358( 6061)     wR2(reflections)= 0.0719( 8904)

S = 0.862                  Npar= 505
```

The following ALERTS were generated. Each ALERT has the format
test-name_ALERT_alert-type_alert-level.
Click on the hyperlinks for more details of the test.

Alert level A

```
PLAT213_ALERT_2_A Atom O10            has ADP max/min Ratio .....        5.8 prolat
```

Alert level B

```
PLAT213_ALERT_2_B Atom O9             has ADP max/min Ratio .....        4.1 prolat
PLAT213_ALERT_2_B Atom O17            has ADP max/min Ratio .....        4.8 prolat
PLAT220_ALERT_2_B Non-Solvent Resd 1   O   Ueq(max)/Ueq(min) Range       8.0 Ratio
PLAT241_ALERT_2_B High   'MainMol' Ueq as Compared to Neighbors of       O18 Check
PLAT242_ALERT_2_B Low    'MainMol' Ueq as Compared to Neighbors of       Fe2 Check
PLAT973_ALERT_2_B Check Calcd Positive Residual Density on      W5      1.58 eA-3
PLAT975_ALERT_2_B Check Calcd Residual Density  0.60A From     O18      1.61 eA-3
PLAT975_ALERT_2_B Check Calcd Residual Density  0.44A From     O10      1.54 eA-3
```

Alert level C

```
PLAT213_ALERT_2_C Atom O3             has ADP max/min Ratio .....        3.5 prolat
PLAT213_ALERT_2_C Atom O5             has ADP max/min Ratio .....        3.1 prolat
PLAT213_ALERT_2_C Atom O7             has ADP max/min Ratio .....        3.5 prolat
PLAT213_ALERT_2_C Atom O11            has ADP max/min Ratio .....        3.8 prolat
PLAT213_ALERT_2_C Atom O12            has ADP max/min Ratio .....        3.8 prolat
PLAT213_ALERT_2_C Atom O13            has ADP max/min Ratio .....        3.3 prolat
PLAT213_ALERT_2_C Atom O14            has ADP max/min Ratio .....        3.1 prolat
PLAT213_ALERT_2_C Atom O16            has ADP max/min Ratio .....        3.7 prolat
PLAT213_ALERT_2_C Atom O18            has ADP max/min Ratio .....        4.0 prolat
PLAT214_ALERT_2_C Atom C15     (Anion/Solvent) ADP max/min Ratio         4.3 prolat
PLAT241_ALERT_2_C High   'MainMol' Ueq as Compared to Neighbors of        O7 Check
PLAT241_ALERT_2_C High   'MainMol' Ueq as Compared to Neighbors of        O8 Check
PLAT241_ALERT_2_C High   'MainMol' Ueq as Compared to Neighbors of        O9 Check
PLAT241_ALERT_2_C High   'MainMol' Ueq as Compared to Neighbors of       O12 Check
PLAT241_ALERT_2_C High   'MainMol' Ueq as Compared to Neighbors of       O13 Check
PLAT241_ALERT_2_C High   'MainMol' Ueq as Compared to Neighbors of       O14 Check
PLAT241_ALERT_2_C High   'MainMol' Ueq as Compared to Neighbors of       O17 Check
PLAT241_ALERT_2_C High   'MainMol' Ueq as Compared to Neighbors of       C13 Check
PLAT241_ALERT_2_C High   'MainMol' Ueq as Compared to Neighbors of       C14 Check
PLAT241_ALERT_2_C High   'MainMol' Ueq as Compared to Neighbors of       C15 Check
PLAT241_ALERT_2_C High   'MainMol' Ueq as Compared to Neighbors of       C17 Check
PLAT241_ALERT_2_C High   'MainMol' Ueq as Compared to Neighbors of       C18 Check
PLAT242_ALERT_2_C Low    'MainMol' Ueq as Compared to Neighbors of        W1 Check
PLAT242_ALERT_2_C Low    'MainMol' Ueq as Compared to Neighbors of        W2 Check
PLAT242_ALERT_2_C Low    'MainMol' Ueq as Compared to Neighbors of        W3 Check
PLAT242_ALERT_2_C Low    'MainMol' Ueq as Compared to Neighbors of        W4 Check
PLAT242_ALERT_2_C Low    'MainMol' Ueq as Compared to Neighbors of        W5 Check
PLAT242_ALERT_2_C Low    'MainMol' Ueq as Compared to Neighbors of        W6 Check
PLAT250_ALERT_2_C Large U3/U1 Ratio for Average U(i,j) Tensor ....       3.0 Note
PLAT342_ALERT_3_C Low Bond Precision on  C-C Bonds ...............    0.0137 Ang.
PLAT413_ALERT_2_C Short Inter XH3 .. XHn     H1M2   ..  H24A    ..      2.11 Ang.
PLAT731_ALERT_1_C Bond    Calc     1.34(2), Rep    1.340(6) ......         3 su-Rat
              C13  -C14         1.555   1.555 ...........     #   84 Check
PLAT731_ALERT_1_C Bond    Calc   1.340(19), Rep    1.340(6) ......         3 su-Rat
              C14  -C15         1.555   1.555 ...........     #   86 Check
PLAT731_ALERT_1_C Bond    Calc   1.371(16), Rep    1.370(5) ......         3 su-Rat
              C16  -C17         1.555   1.555 ...........     #   89 Check
PLAT906_ALERT_3_C Large K value in the Analysis of Variance ......     4.368 Check
PLAT910_ALERT_3_C Missing # of FCF Reflection(s) Below Theta(Min)          8 Note
PLAT911_ALERT_3_C Missing # FCF Refl Between THmin & STh/L=  0.600         3 Report
```

```
PLAT971_ALERT_2_C Check Calcd Residual Density  0.75A From      W1       2.44 eA-3
PLAT971_ALERT_2_C Check Calcd Residual Density  0.96A From      W6       2.31 eA-3
PLAT971_ALERT_2_C Check Calcd Residual Density  0.86A From      W4       2.19 eA-3
PLAT971_ALERT_2_C Check Calcd Residual Density  0.40A From      W4       2.16 eA-3
PLAT971_ALERT_2_C Check Calcd Residual Density  0.23A From      W1       2.14 eA-3
PLAT971_ALERT_2_C Check Calcd Residual Density  0.92A From     O16       2.06 eA-3
PLAT971_ALERT_2_C Check Calcd Residual Density  0.90A From      W6       2.05 eA-3
PLAT971_ALERT_2_C Check Calcd Residual Density  0.81A From      W2       1.88 eA-3
PLAT971_ALERT_2_C Check Calcd Residual Density  0.92A From      W5       1.83 eA-3
PLAT971_ALERT_2_C Check Calcd Residual Density  1.02A From      W3       1.81 eA-3
PLAT971_ALERT_2_C Check Calcd Residual Density  0.92A From      W3       1.80 eA-3
PLAT971_ALERT_2_C Check Calcd Residual Density  0.95A From      W1       1.73 eA-3
PLAT971_ALERT_2_C Check Calcd Residual Density  0.96A From      O9       1.72 eA-3
PLAT971_ALERT_2_C Check Calcd Residual Density  0.99A From      W5       1.64 eA-3
PLAT971_ALERT_2_C Check Calcd Residual Density  0.90A From      W4       1.62 eA-3
PLAT971_ALERT_2_C Check Calcd Residual Density  0.60A From     O18       1.61 eA-3
PLAT971_ALERT_2_C Check Calcd Residual Density  1.04A From      W3       1.59 eA-3
PLAT971_ALERT_2_C Check Calcd Residual Density  0.94A From     O16       1.58 eA-3
PLAT971_ALERT_2_C Check Calcd Residual Density  0.80A From      W1       1.57 eA-3
PLAT971_ALERT_2_C Check Calcd Residual Density  0.63A From      W6       1.55 eA-3
PLAT971_ALERT_2_C Check Calcd Residual Density  0.86A From      W5       1.55 eA-3
PLAT971_ALERT_2_C Check Calcd Residual Density  0.84A From      W2       1.55 eA-3
PLAT971_ALERT_2_C Check Calcd Residual Density  0.44A From     O10       1.54 eA-3
PLAT972_ALERT_2_C Check Calcd Residual Density  0.83A From      W1      -2.20 eA-3
PLAT975_ALERT_2_C Check Calcd Residual Density  0.63A From     O11       1.50 eA-3
PLAT977_ALERT_2_C Check the Negative Difference Density on    H1M1      -0.49 eA-3
PLAT977_ALERT_2_C Check the Negative Difference Density on      H2      -0.36 eA-3
PLAT977_ALERT_2_C Check the Negative Difference Density on    H1M2      -0.43 eA-3
PLAT977_ALERT_2_C Check the Negative Difference Density on    H1M3      -0.76 eA-3
PLAT977_ALERT_2_C Check the Negative Difference Density on      H4      -0.32 eA-3
PLAT977_ALERT_2_C Check the Negative Difference Density on      H7      -0.31 eA-3
PLAT977_ALERT_2_C Check the Negative Difference Density on     H11      -0.46 eA-3
PLAT977_ALERT_2_C Check the Negative Difference Density on     H19      -0.35 eA-3
PLAT977_ALERT_2_C Check the Negative Difference Density on    H21A      -0.40 eA-3
PLAT978_ALERT_2_C Number C-C Bonds with Positive Residual Density.           0 Note
```

Alert level G

```
PLAT003_ALERT_2_G Number of Uiso or Uij Restrained non-H Atoms ...          30 Report
PLAT007_ALERT_5_G Number of Unrefined Donor-H Atoms ..............           1 Report
PLAT042_ALERT_1_G Calc. and Reported MoietyFormula Strings  Differ     Please Check
PLAT177_ALERT_4_G The CIF-Embedded .res File Contains DELU Records           6 Report
PLAT186_ALERT_4_G The CIF-Embedded .res File Contains ISOR Records           1 Report
PLAT300_ALERT_4_G Atom Site Occupancy of *O19    is Constrained at         0.5 Check
PLAT300_ALERT_4_G Atom Site Occupancy of *O20    is Constrained at         0.5 Check
PLAT300_ALERT_4_G Atom Site Occupancy of *O21    is Constrained at         0.5 Check
PLAT300_ALERT_4_G Atom Site Occupancy of *O22    is Constrained at         0.5 Check
PLAT301_ALERT_3_G Main Residue  Disorder .............(Resd  1)..           8 % Note
PLAT302_ALERT_4_G Anion/Solvent/Minor-Residue Disorder (Resd  2)..          91 % Note
PLAT432_ALERT_2_G Short Inter X...Y Contact  O3     ..  C5      ..        2.98 Ang.
PLAT710_ALERT_4_G Delete 1-2-3 or 2-3-4 Linear Torsion Angle ... #         125 Do !
            O19 -SI1 -O19 -W3      3.00  0.00   2.656   1.555   1.555   1.555
PLAT710_ALERT_4_G Delete 1-2-3 or 2-3-4 Linear Torsion Angle ... #         132 Do !
            O19 -SI1 -O19 -W2     15.00  0.00   2.656   1.555   1.555   1.555
PLAT710_ALERT_4_G Delete 1-2-3 or 2-3-4 Linear Torsion Angle ... #         139 Do !
            O19 -SI1 -O19 -W1      9.00  0.00   2.656   1.555   1.555   1.555
PLAT710_ALERT_4_G Delete 1-2-3 or 2-3-4 Linear Torsion Angle ... #         197 Do !
            O20 -SI1 -O20 -O21    13.00  0.00   2.656   1.555   1.555   2.656
PLAT710_ALERT_4_G Delete 1-2-3 or 2-3-4 Linear Torsion Angle ... #         204 Do !
            O20 -SI1 -O20 -W5     13.00  0.00   2.656   1.555   1.555   1.555
PLAT710_ALERT_4_G Delete 1-2-3 or 2-3-4 Linear Torsion Angle ... #         211 Do !
```

```
            O20 -SI1 -O20 -W2      0.00  0.00   2.656   1.555   1.555   1.555
PLAT710_ALERT_4_G Delete 1-2-3 or 2-3-4 Linear Torsion Angle ... #       218 Do !
            O20 -SI1 -O20 -W6     12.00  0.00   2.656   1.555   1.555   1.555
PLAT710_ALERT_4_G Delete 1-2-3 or 2-3-4 Linear Torsion Angle ... #       294 Do !
            O21 -SI1 -O21 -O20    13.00  0.00   2.656   1.555   1.555   2.656
PLAT710_ALERT_4_G Delete 1-2-3 or 2-3-4 Linear Torsion Angle ... #       300 Do !
            O21 -SI1 -O21 -W3      1.00  0.00   2.656   1.555   1.555   1.555
PLAT710_ALERT_4_G Delete 1-2-3 or 2-3-4 Linear Torsion Angle ... #       307 Do !
            O21 -SI1 -O21 -W5     13.00  0.00   2.656   1.555   1.555   2.656
PLAT710_ALERT_4_G Delete 1-2-3 or 2-3-4 Linear Torsion Angle ... #       314 Do !
            O21 -SI1 -O21 -W4     11.00  0.00   2.656   1.555   1.555   1.555
PLAT710_ALERT_4_G Delete 1-2-3 or 2-3-4 Linear Torsion Angle ... #       375 Do !
            O22 -SI1 -O22 -W4      5.00  0.00   2.656   1.555   1.555   1.555
PLAT710_ALERT_4_G Delete 1-2-3 or 2-3-4 Linear Torsion Angle ... #       382 Do !
            O22 -SI1 -O22 -W6      8.00  0.00   2.656   1.555   1.555   1.555
PLAT710_ALERT_4_G Delete 1-2-3 or 2-3-4 Linear Torsion Angle ... #       389 Do !
            O22 -SI1 -O22 -W1     17.00  0.00   2.656   1.555   1.555   2.656
PLAT720_ALERT_4_G Number of Unusual/Non-Standard Labels ..........         3 Note
PLAT860_ALERT_3_G Number of Least-Squares Restraints .............       120 Note
PLAT912_ALERT_4_G Missing # of FCF Reflections Above STh/L=  0.600         6 Note
```

---

  1 **ALERT level A** = Most likely a serious problem - resolve or explain
  8 **ALERT level B** = A potentially serious problem, consider carefully
 72 **ALERT level C** = Check. Ensure it is not caused by an omission or oversight
 29 **ALERT level G** = General information/check it is not something unexpected

  4 ALERT type 1 CIF construction/syntax error, inconsistent or missing data
 76 ALERT type 2 Indicator that the structure model may be wrong or deficient
  6 ALERT type 3 Indicator that the structure quality may be low
 23 ALERT type 4 Improvement, methodology, query or suggestion
  1 ALERT type 5 Informative message, check

It is advisable to attempt to resolve as many as possible of the alerts in all categories. Often the minor alerts point to easily fixed oversights, errors and omissions in your CIF or refinement strategy, so attention to these fine details can be worthwhile. In order to resolve some of the more serious problems it may be necessary to carry out additional measurements or structure refinements. However, the purpose of your study may justify the reported deviations and the more serious of these should normally be commented upon in the discussion or experimental section of a paper or in the "special_details" fields of the CIF. checkCIF was carefully designed to identify outliers and unusual parameters, but every test has its limitations and alerts that are not important in a particular case may appear. Conversely, the absence of alerts does not guarantee there are no aspects of the results needing attention. It is up to the individual to critically assess their own results and, if necessary, seek expert advice.

Publication of your CIF in IUCr journals

A basic structural check has been run on your CIF. These basic checks will be run on all CIFs submitted for publication in IUCr journals (*Acta Crystallographica*, *Journal of Applied Crystallography*, *Journal of Synchrotron Radiation*); however, if you intend to submit to *Acta Crystallographica Section C* or *E* or *IUCrData*, you should make sure that full publication checks are run on the final version of your CIF prior to submission.

Publication of your CIF in other journals

Please refer to the *Notes for Authors* of the relevant journal for any special instructions relating to CIF submission.

PLATON version of 24/11/2016; check.def file version of 23/11/2016

Datablock am81_100 - ellipsoid plot

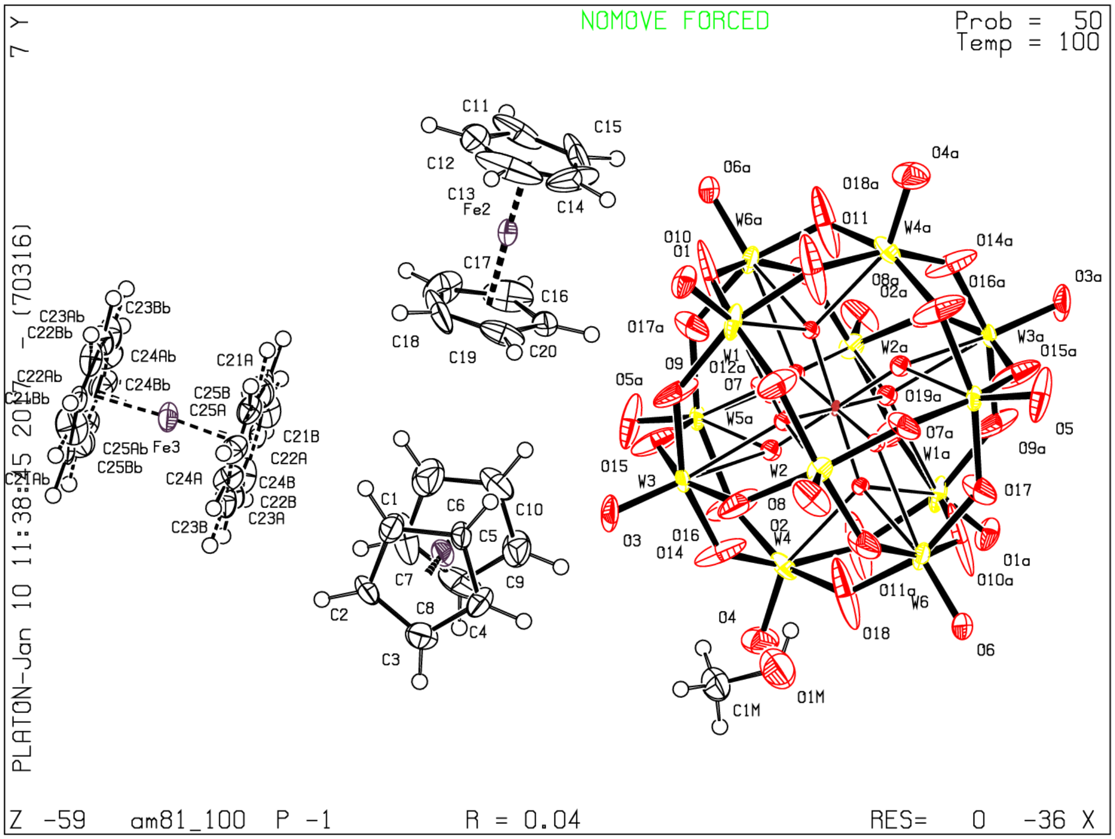

Supplement: Supplementary file 1 [file molecules-23-03150-s001.pdf]
